# Insights into DNA hydroxymethylation in the honeybee from in-depth analyses of TET dioxygenase

**DOI:** 10.1098/rsob.140110

**Published:** 2014-08-06

**Authors:** Marek Wojciechowski, Dominik Rafalski, Robert Kucharski, Katarzyna Misztal, Joanna Maleszka, Matthias Bochtler, Ryszard Maleszka

**Affiliations:** 1 Laboratory of Structural Biology, International Institute of Molecular and Cell Biology, 02-109 Warsaw, Poland; 2 Research School of Biology, The Australian National University, Canberra, Australian Capital Territory 0200, Australia

**Keywords:** epigenetic code, epigenomics, brain plasticity, phenotypic polymorphism, demethylation, social insect

## Abstract

In mammals, a family of TET enzymes producing oxidized forms of 5-methylcytosine (5mC) plays an important role in modulating DNA demethylation dynamics. In contrast, nothing is known about the function of a single TET orthologue present in invertebrates. Here, we show that the honeybee TET (AmTET) catalytic domain has dioxygenase activity and converts 5mC to 5-hydroxymethylcytosine (5hmC) in a HEK293T cell assay. *In vivo*, the levels of 5hmC are condition-dependent and relatively low, but in testes and ovaries 5hmC is present at approximately 7–10% of the total level of 5mC, which is comparable to that reported for certain mammalian cells types. AmTET is alternatively spliced and highly expressed throughout development and in adult tissues with the highest expression found in adult brains. Our findings reveal an additional level of flexible genomic modifications in the honeybee that may be important for the selection of multiple pathways controlling contrasting phenotypic outcomes in this species. In a broader context, our study extends the current, mammalian-centred attention to TET-driven DNA hydroxymethylation to an easily manageable organism with attractive and unique biology.

## 2. Background

Epigenomic modifications of sundry types are important components of multi-factorial molecular machinery controlling cellular responses to a wide range of factors, both internal and external. These flexible alterations to DNA and chromatin via methylation and demethylation processes, as well as by reversible histone modifications, act as a degenerate ‘epigenetic code’ [1] that participates in regulatory networks controlling context-dependent gene expression [2,3]. One type of DNA modification that is of special interest consists of chemical marks on genomic cytosines [4,5]. In mammals, cytosine methylation is catalysed by type 1 and 3 methyltransferases (DNMTs 1 and 3), whereas methylation erasure is mediated by the family of TET dioxygenases (TET 1–3) that convert 5-methylcytosine (5mC) to 5-hydroxymethylcytosine (5hmC), 5-formyl-cytosine (5fC) and 5-carboxyl-cytosine (5caC) [6,7]. Initially, hydroxylation of 5mCs was considered an intermediate step in a DNA demethylation pathway required for the high level of flexibility underlying epigenetic gene regulation in development, brain plasticity, genomic imprinting and transcriptional changes induced by environmental insults [6,8]. However, recent findings including genome-wide mapping of 5hmC at a single-base resolution in mammalian brain reveal a more complex picture consistent with the idea that both 5mC and 5hmC can act as independent epigenetic marks [9–11]. The notion of 5hmC and 5mC being discrete epigenomic modifiers is supported by the recent study showing that dynamic readers for both bases are only partly overlapping and some readers display clear-cut specificities for only one of them [12].

Although there are notable differences in the number of genes encoding DNMT1 and DNMT3s in various metazoan species, including the lack of these enzymes in *Diptera* and in certain nematodes, the basic properties of the DNA methylation toolkit, including the preferred specificity for cytosines occurring in the CpG context, appear to be conserved throughout the animal lineage [13,14]. In contrast, it is not clear whether an active demethylation pathway similar to that operating in mammals exists in non-mammalian organisms. So far, only single TET relatives with unspecified catalytic activities have been found in the majority of sequenced invertebrate genomes including several insect species [7]. TET proteins are absent in organisms that have lost the entire DNA methylation toolkit, such as *Caenorhabditis elegans*. However, the presence of TET in *Drosophila melanogaster*, which only has a t-RNA methylating enzyme DNMT2 [15], suggests that TET activity in invertebrates may not be restricted to DNA templates.

The growing importance of insect models, in particular the honeybee, in epigenetic research prompted us to determine whether the single TET protein in this species has the capacity to convert 5mC to 5hmC. *Apis mellifera* already is a prominent system for methylomics and an emerging model for histone research [16–18]. Its striking nutritionally controlled development combined with adult behavioural plasticity and haplodiploidy of sex determinations offers a formidable biological setting for epigenetic studies. Here, we present a seminal in-depth characterization of an invertebrate TET at the biochemical and molecular level.

## 3. Results

### 3.1. Detection of 5hmC in *Apis mellifera*

**Figure 1.**
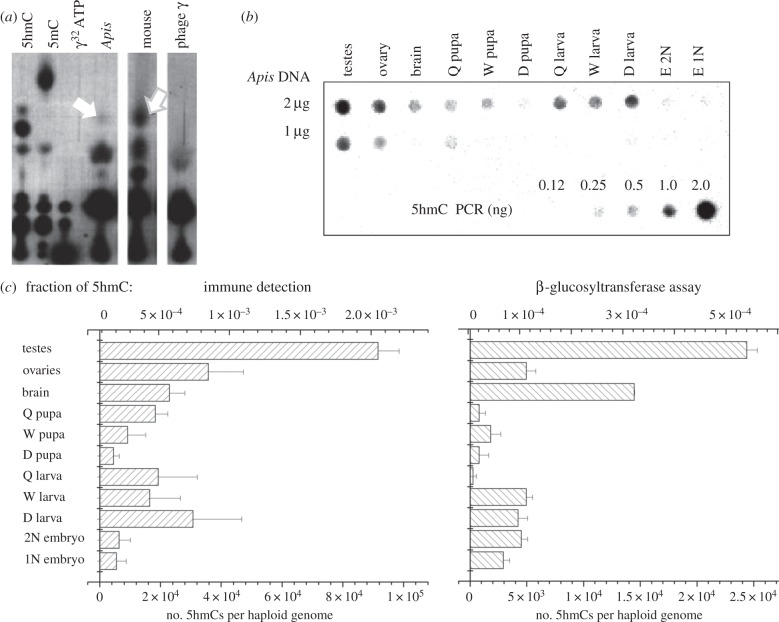
(*a*) TLC identification of 5hmC in *A. mellifera.* Single radiolabelled nucleotides derived from a variety of samples were separated by TLC on PEI cellulose. Samples obtained from single dNTPs were used as standards. Three genomic DNA preparations were digested to single nucleotides and immunoprecipitated with anti-5hmC antibodies. Purified nucleotides were radiolabelled and resolved by TLC. A spot representing 5hmC is present in drone testes DNA, whereas a much stronger spot can be seen for mouse brain known to be enriched in 5hmC (white arrows). No 5hmC spot can be found in *λ* phage (Dam^−^ Dcm^−^). All lanes are from the same TLC plate. (*b*) An image of a DNA dot-blot hybridized with an anti-5hmC antibody. For each sample, 1 and 2 µg of DNA were spotted on the membrane. A PCR product with dCTP substituted for d5hmCTP was used as control. (*c*) 5hmC quantification in various honeybee DNA samples. The data points were obtained using two methods; a densitometry scan of the dot-blots shown in (*b*), and a β-glucosyltransferase assay (see Material and methods). The resulting values were plotted as either fractions of total cytosines (top) or as a number of 5hmCs in a haploid genome (bottom). The overall correlation between two methods is 0.712. Q, W, D and E refer to queen, worker, drone and embryos, respectively.

We have used three methods to show the presence of 5hmC *in vivo*. First, by using thin layer chromatography (TLC), we have confirmed that a spot at the position expected for 5hmC is detectable in DNA samples extracted from *A. mellifera* (figure 1*a*). In comparison with the mouse brain DNA, the intensity of the honeybee signal is much weaker, most likely reflecting the two orders of magnitude lower levels of CpG methylation [13,17] and hydroxymethylation (see below) in this species. Next, we have used the immunoblot assay to estimate 5hmC levels in different castes, tissues and developmental stages. As a reference, we used PCR products that were made with 5hmC nucleoside triphosphate instead of the usual cytidine triphosphate (CTP). As shown in figure 1*b,c*, 5hmC is detectable in all examined samples with the highest levels found in drone testes and queen ovaries. As in mammals, 5hmC levels appear to be relatively high in brain compared with other tissues. Very low levels of 5hmC are present during metamorphosis in pupae of all three castes (workers, queen, drones) and in the hypopharyngeal gland (not shown), whereas both haploid and diploid embryos show low to moderate levels of 5hmC. We also note that the variation in the amount of 5hmC in *A. mellifera* genomic DNA samples appears to be larger than the variation that would be expected from DNA damage. Although control experiments have established that the 5hmC antibody does not significantly cross-react with 5mC or cytosine, we cannot rule out the possibility that it detects unknown antigens other than 5hmC in our samples. Moreover, the efficiency of the antibody is known to depend on the density of 5hmC [9]. To obtain further evidence, we have conducted additional 5hmC quantifications using an alternative approach, namely the glucosyltransferase assay that is not affected by these limitations. The T4-glucosyltransferase assay depends on the transfer of radioactively labelled glucose from UDP-glucose to 5hmC. Control experiments have shown that transfer of glucose to 5-hydroxymethyluracil, a base that could be present owing to oxidative damage to thymines, is undetectable (not shown), suggesting that the assay exclusively quantifies 5hmC. The results are shown in figure 1*c*. From the comparison with the calibration curve, we estimate that the highest number of 5hmCs in the *A. mellifera* genomic samples is approximately 7000 per haploid genome (figure 1*c*) and thus is at least two to three orders of magnitude lower than the total number of 5hmCs per haploid genome in mammals [7,11]. The results generated using the immune blots and glucosyltransferase assay (figure 1*c*) are in excellent agreement with the overall correlation between the two methods, calculated to be 0.712. Furthermore, both the T4-glucosyltransferase assay and immunoblots have been conducted twice on separate biological materials collected from different colonies a few months apart yielding similar results.

### 3.2. Cloning and *in vitro* expression of AmTET

**Figure 2.**
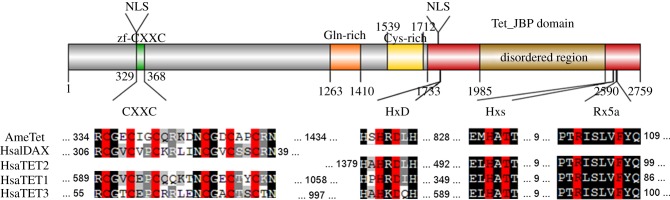
Domain organization of *A. mellifera* TET. The core catalytic region of AmTET is located at the C-terminus. It consists of a Cys-rich domain followed by an iron (II)–oxoglutarate-dependent dioxygenase domain (Tet–JBP). This domain harbours an intrinsically disordered 600 amino acid insertion. Known signature motives of Tet–JBP domains from *A. mellifera* and human TETs are aligned below the domain organization diagram, with critical residues highlighted in red. These motives are: HxD and Hxs (where s is a small residue) responsible for iron coordination and Rx5a (where a is an aromatic residue) responsible for 2-oxo acid coordination. The gene model available via BeeBase (www.beebase.org) does not have an extra exon coding for 25 amino acids and a few mini exons (figure 5) that we found by examining RNAseq datasets. Accession numbers: HsaTET1, NP_085128.2; HsaTET2, NP_001120680.1; HsaTET3, XP_005264244.1; HsaIDAX, NP_079488.2; AmeTET, GB52555 (BeeBase OGSv3.2).

**Figure 3.**
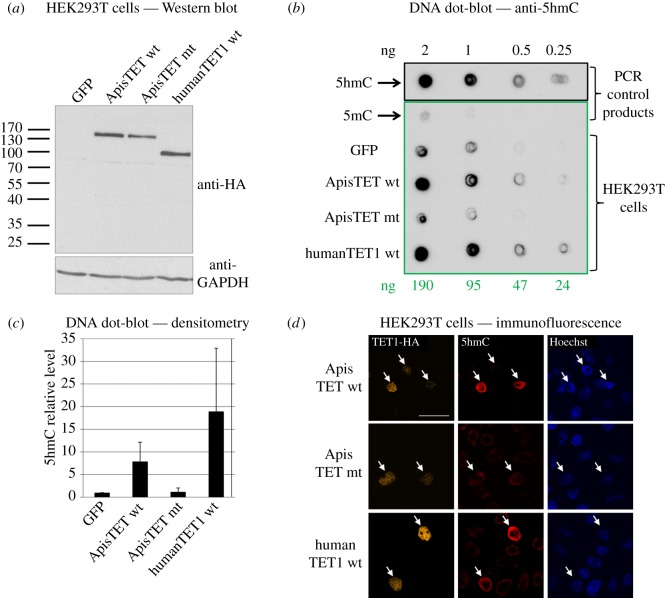
*In vitro* expression of AmTET in human embryonic kidney (HEK293T) cells. (*a*) Western blot analysis of the expression levels of TET-HA proteins were analysed 48 h after transfection. GAPDH level is a protein loading control. (*b*) Genomic DNA from these cells was isolated and dot-blotted with specific anti-5hmC antibody. About 24–190 ng of each genomic DNA and the same amounts of PCR product with dCTP swapped for d5mCTP were used for this experiment. About 0.25–2ng of PCR product with dCTP swapped for d5hmCTP was used as a positive control. Signal obtained from cells expressing wild-type TETs is stronger than from cells expressing GFP or a catalytically inactivated honeybee TET. (*c*) Data obtained from three independent dot-blots were quantified by densitometry. Results were normalized with DNA obtained from cells expressing GFP (set as 1). (*d*) TET localization and 5hmC presence in transfected HEK293T cells was analysed via immunofluorescence. HA-tagged TET proteins (orange) localize mainly in the nuclei (blue). Cells that express catalytically competent TETs also have more 5hmC (red). The increase of hydroxymethylation in transfected cells is statistically significant (the *p-*value of the null hypothesis is 0.054). Scale bar, 20 µm. The arrows point to cells with TETs. The image represents a single slice of a confocal stack. The ring for 5hmC staining is expected because of the penetration depth of denaturation affecting the 5hmC detection. In contrast, both TET detection and DAPI staining do not require denaturation of the DNA, and hence do not show the same ring feature. Although HEK293 cells have very low endogenous TET and 5hmC, their residual amounts result in a small background.

By BLAST searching the honeybee genome with mammalian TET proteins, we have identified a large gene, greater than 150 kb, encoding a predicted polypeptide harbouring all the signature domains and motifs characteristic of the TET oxoglutarate-dependent dioxygenase protein family, namely the HxD and Hxs motifs implicated in Fe(II) binding, the oxoglutarate recognition signature Rx5a and the DNA-binding CXXC domain [7]. Like in mammalian TETs, the honeybee protein has a Cys-rich region located upstream of the catalytic domain and a long amino acid insertion. To demonstrate that the putative AmTET protein has dioxygenase activity, we have cloned its C-terminal fragment, spanning the catalytic domain, the Cys-rich domain and a predicted nuclear localization signal (figure 2). We then expressed AmTET in human embryonic kidney (HEK293T) cells and monitored the levels of 5hmC using both dot-blots and immunofluorescence imaging. *In vitro* expression of AmTET was carried out in HEK293T cells that contain low endogenous 5hmC levels, but have ample 5mC to provide the substrate for oxidation. The AmTET fragment with amino-terminal haemagglutinin (HA) tag was placed under the control of the CMV promoter and transfected into HEK293T cells. In order to ensure that any observed effects were due to the catalytic activity of the AmTET, we used as the negative control cells transfected with a GFP-coding plasmid and a predicted catalytically inactive mutant of AmTET. The inactive AmTET double mutant has the sequence YxA instead of HxD, because this substitution was previously shown to inactivate the mammalian TET1 [19]. As a positive control, we have used a previously described HA-tagged fragment of human TET1, placed in the same vector with the CMV promoter. The expression levels of TET proteins and the abundance of 5hmC were analysed after 48 h or after 16 h upon proteasome inhibition. Protein levels were measured with anti-HA-tag antibody and turned out to be similar for all three constructs. The 5hmC levels were monitored by the dot-blot assay using commercially available anti-5hmC antibody. As expected, we have detected a five to 20-fold increase of 5hmC levels in cells expressing the wild-type honeybee or human TET, relative to cells expressing GFP or mutant TET incapable of iron binding (figure 3). Given the apparent nuclear localization of AmTET in HEK293T cells (figure 3*d*), the increase in 5hmC levels has to result from the conversion of 5mCs present in nuclear DNA.

### 3.3. Transcriptional profiling of AmTET

**Figure 4.**
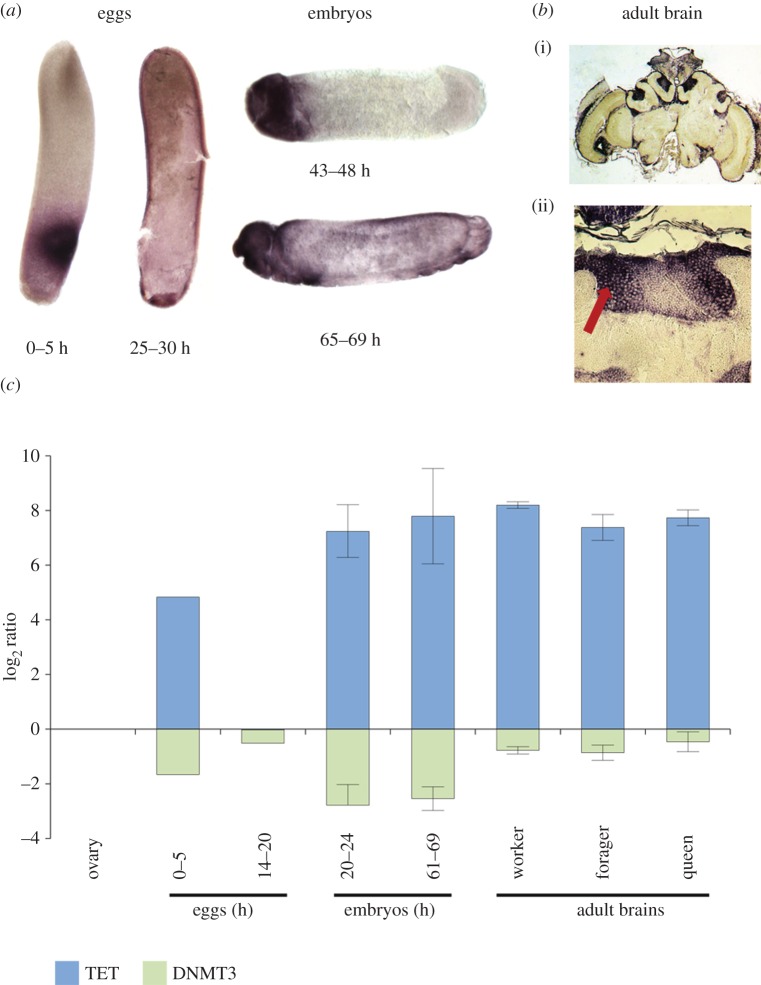
*In vivo* expression of AmTET. (*a*) *In situ* hybridization showing the localization of AmTET transcripts in eggs and during embryogenesis. (*b*) *In situ* hybridization showing the localization of AmTET transcripts in adult brain (nurse bee): (i) whole brain; (ii) high magnification of one calyx. The red arrow indicates the area occupied by large Kenyon cells. Control hybridizations with sense probe detect no signal (not shown). (*c*) qPCR analysis of AmTET and AmDNMT3 expression in eggs, embryos and adult brains, relative to ovarian expression of both genes.

Using both qPCR and *in situ* hybridization, we have examined the expression of AmTET during early development and in adult tissues. AmTET transcripts are relatively abundant in 0–5 h eggs, but scarce during early/mid-blastoderm formation (14–20 h; figure 4*a*,*c*). Because 0–5 h eggs contain only four to seven nuclei, a relatively high level of any transcript at this stage is considered to be of maternal origin. The maternal transcripts are eliminated from the embryo at the midblastula transition [20,21] and are replaced by zygotic transcription that already is detectable at late blastoderm formation phase (25–30 h, figure 4*a*). From the germ band stage (approx. 40–48 h) until the completion of larval body at the pre-hatching phase (69–72 h), the levels of AmTET are relatively high especially in the nervous system that begins to form around 40 h (figure 4*a*). The expression levels in adult brains are comparable to those in late embryos and are very similar in foragers, nurses and mated egg-laying queens (figure 4*c*). Although AmTET appears to be ubiquitously expressed in most or all brain cells (figure 4*b*(i)), a higher magnification of the mushroom body calyces reveals a distinct pattern indicative of a preferential expression of this gene in large Kenyon cells (red arrow in figure 4*b*(ii)) whose somata are located at the inside edges of the calyces [21].

To compare the relative levels of AmTET and a putative de novo DNA methyltransferase, AmDNMT3, we have contrasted their expression during early development and in adult brains. As shown in figure 4*c*, AmTET and AmDNMT3 are expressed in all examined situations, but AmTET transcripts are by far more abundant than AmDNMT3 transcripts. Finally, we have surveyed our extensive RNAseq datasets for the presence of AmTET transcripts in other samples and to compare the expression of AmTET with other genes (electronic supplementary material, figures S1 and S2). In addition to embryos, ovaries and brains, AmTET is also expressed in larvae and antennae (electronic supplementary material, figures S1 and S2), testes and pupae (electronic supplementary material, figure S3). In good agreement with the *in situ* hybridization and qPCR, the highest number of AmTET transcripts is found in libraries from adult brains with larval quantities found to be in the range of 5–10 times lower. Interestingly, larval expression differs among the three distinct castes, with the drone larvae dataset having the lowest numbers of AmTET transcripts and the worker larvae the highest. In the brain, AmTET is one of the 1000 most expressed genes (approx. 6% of all genes; electronic supplementary material, figure S1). The level of AmTET expression in other tissues/stages is variable, but there appears to be an approximate positive correlation between the transcript abundance and the quantities of detected 5hmC. One exception is the queen ovary, which shows a very low level of expression in spite of being relatively rich in 5hmC (figure 1 and electronic supplementary material, figure S3).

Although at this stage the reason for this result is unclear, one possibility is that highly polyploid trophocytes (nurse cells) that contribute the majority of RNA (approx. 90%) extracted from ovaries have low levels of AmTET and dilute the signal from oocytes.

### 3.4. Alternative transcripts variants of AmTET

**Figure 5.**
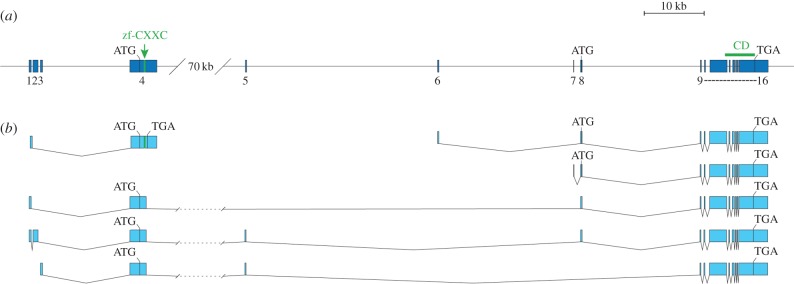
AmTET gene model and transcript variants. (*a*) Manually annotated gene model showing all detected exons. zf-CXXC, DNA-binding domain; CD, catalytic domain. (*b*) Selected transcript models based on RNAseq data. Based on gene assembly OGSv3.2 (www.beebase.org), TET id: GB52555. Genomic location: linkage group LG12 (NCBI reference sequence: NC_007081.3), nucleotides 4 499 630–4 665 644. For more detail on transcript variants detection, see the electronic supplementary material, figure S4.

The existence of a single TET orthologue in diverse invertebrates [7], including a cnidarian *Hydra magnipapillata* [22], suggests that this gene originated early in metazoan evolution and encoded a mosaic protein with all key signatures found in present-day TETs. Later, duplications in vertebrates generated two protein variants with or without the CXXC DNA-binding module, which is not required for hydroxylation of 5mC but is important for context-dependent activities of TETs [7,23]. Interestingly, recent experiments have uncovered important functional features of CXXC modules in vertebrate TETs; first, the CXXC domains in TET1 and TET3 have distinct binding properties [23], and second, the alternative isoforms of TETs can interconnect with distinct modules belonging to the CXXC zinc finger family [22]. Acting together with the catalytic domain, various CXXC modules expand the repertoire of TET-mediated target gene regulation [23]. These reports prompted us to investigate if there is a splicing pattern of AmTET that could generate variants with different combination of modules. Our analyses of the available RNAseq data show that that the majority of AmTET transcripts (approx. 80%) span the upstream exon encoding the CXXC zinc finger (figure 5*a,b* and the electronic supplementary material, figure S4). Although the three CXXC-plus variants have different 5′ ends (exon 1, 1 + 2 or 3, respectively) and a distinct combination of micro-exons 5 and 8 (figure 5*a,b*), they code for identical proteins with respect to both the catalytic and DNA-binding domain. However, CXXC-minus mRNAs with alternate 5′-ends (micro-exons 6 and 7; figure 5) are also produced and, moreover, around 10% of transcripts from this genomic region encode a stand-alone CXXC module (figure 5*b*). This intricate pattern of expression suggests that other alternatively spliced transcripts not detected by this approach may be generated in a combinatorial manner. Whether this transcriptional complexity indicates a coding potential for additional AmTET protein isoforms with expanded connectivity to various cellular pathways needs to be addressed by further experiments.

## 4. Discussion

Our data clearly indicate that a single TET orthologue in *A. mellifera* can oxidase 5mC to 5hmC. Overexpression of AmTET in mammalian HEK293T cells significantly increases the level of 5hmC, which is consistent with the oxygen- and oxoglutarate-dependent reaction generally accepted for mammalian TETs. 5hmC also is detectable *in vivo* both during development and in adult stages. We assume that all 5hmCs detectable in *A. mellifera* result from TET-dependent oxidation of 5mC in genomic DNA, because alternative pathways, such as the reaction of cytosine with formaldehyde, have never been demonstrated *in vivo* [24]. The total amount of 5hmCs found in *A. mellifera* is predictably low in accord with the current view that TET dioxygenases catalyse the synthesis of 5hmC from 5mC. In contrast to over 20 million 5mCs in the mammalian genome [5], there are only approximately 70 000 5mCs identified by genome-wide methylomics in adult brains and larval heads of worker bees [16,17], which occur predominantly in the CpG context in intragenic regions of approximately 6000 conserved nuclear genes (no DNA methylation has been detected in *A. mellifera* mitochondrial DNA [16]). In total, there are approximately only a few thousands 5hmCs per haploid genome in *A. mellifera* compared with a few million 5hmCs found in various mammalian cells. However, when expressed as the fraction of methylated cytosines, the levels of 5hmC in *A. mellifera* appear to be comparable to those in mammals. In testes and ovaries, 5hmCs account for 7–10% of the methylated CpGs, whereas in foetal mammalian brain and in embryonic stem cells 5hmCs account for 10% and 5–10% of all methylated CpGs, respectively [7,11,19]. In other mammalian cells such as some immune cell populations from myeloid malignancies, the fraction of 5hmC is even lower, e.g. 1% [25]. One exception is adult brain where the level of 5hmCs is as high as 40% of the total level of 5mC [26]. Our results have been reassuringly consistent between two independent rounds of experiments performed a few months apart on separate biological materials. Given the low level of 5hmC in *Apis*, such reproducibility implies a rather precise mode of action of the relevant enzymology that maintains context-dependent 5hmC profiles, most likely as part of a process the regulates normal DNA methylation levels and gene expression.

AmTET is expressed in adult tissues and throughout development with the highest level of transcription observed in the central brain, where it belongs to the 6% most abundant mRNAs. Although the patterns of AmTET expression and the observed amounts of 5hmCs are largely positively correlated, the strikingly high abundance of AmTET transcripts, particularly in adult brain, testes and embryonic nervous system, is difficult to reconcile with the scarcity of 5hmC in this species. One possibility is that AmTET performs other functions not related to oxidation of 5mC in line with the emerging role of mammalian TETs in cooperative regulation of gene networks [7,23]. Alternatively, such elevated levels of AmTET in certain situations are needed to drive the high dynamics of methylation–demethylation processes, such as rapid conversion of 5mC to 5hmC owing to environmental stress. In mammals, neuronal TET1 regulates normal DNA methylation levels and in a TET1 knock-out mouse several neuronal activity-dependent genes have been found to be downregulated [27], leading to abnormal brain functions. This effect has been correlated with promoter hypermethylation of a key regulatory gene, *Npas4* [27]. The issue of reformatting methylation marks is of great interest in the context of a hypothetical role that epigenomic modifications may play in gene regulation pertinent to the reproductive interests of males and females in Hymenoptera and the so-called social conflicts [28,29]. In mammals, parental genome demethylation is catalysed by TET3, the only TET expressed at substantial levels in zygotes, whereas TET1 and TET2 are most highly expressed in primordial germ cells (embryonic days 8.5 till 13.5, reviewed in Pastor *et al* [7]). The new methylation patterns are re-established at the blastocyst stage [30] and correlate with high levels of TET1 and TET2 [7]. Our findings suggest some similarities between the transcriptions of AmTET and mammalian TETs during early developmental stages. The profile of AmTET mRNA abundance in embryos shows a medium level of early transcripts followed by very low or no transcription until the late blastoderm stage, from which time point the gene's activity remains high until completion of embryonic development. Whether the embryonic expression pattern of AmTET reflects a demethylation–methylation cycle resembling that in mammals needs to be examined in more detail at the whole genome level.

Evidence in mammals supports a dual role of 5hmC in demethylation, both active [31] and passive [6,32], and as a stable DNA base [7]. In particular, the high level of brain 5hmCs is considered a potential source of meaningful information for brain differentiation [10,11]. In view of the mammalian findings, the patterns of AmTET transcriptional activity in adult brains and embryonic nervous system may be indicative of 5hmC significance in controlling certain brain functions. However, at this stage, we have insufficient data to make definite calls on the meaning of DNA hydroxymethylation in *A. mellifera* in the context of the primary gene regulatory networks. Phenotypic consequences of TET1–3 deficiencies in mice strongly depend not only on which paralogue has been knocked-out but also on the genetic background [7], suggesting that each protein provides a different input into an organism's biology by interacting with distinct signalling networks. Because phenotypic prediction is not automatically derivable from a catalytic protein function, the extent to which the biological significance of the single TET protein in *A. mellifera* is comparable to the role of mammalian TETs needs to be considered with caution.

Some of these important issues can be addressed in *A. mellifera* by high-resolution mapping of 5hmCs in different contexts and by silencing AmTET during embryonic and larval development. The most rewarding outcomes from such manipulations are likely to be in the area of queen/worker nutritionally controlled phenotypic polymorphism, which offers unparalleled insights into epigenetics of developmental canalization and metaboloepigenetics. Furthermore, interference with TET expression in embryos will help to resolve the significance of the hypothesized demethylation dynamics of parental 5mC patterns in the context of male/female haplodiploidy. Evidence for the involvement of mammalian TETs in erasure of genomic imprinting comes from a recent study showing that TET1 is an important player in establishing epigenetic signatures via the removal of genomic methylation marks, including imprinted genes, at the late reprogramming stage [33]. Finally, the small and manageable number of 5hmCs in the adult brain combined with behavioural flexibility of adult workers can be explored to study the role(s) of 5hmC in neuronal plasticity. Our discovery of a conserved hydroxymethylation enzymology in *A. mellifera* greatly expands the value of this organism as a prominent invertebrate model for epigenomic research.

## 5. Material and methods

### 5.1. AmTET cloning and mutagenesis

A synthetic gene of *Homo sapiens* TET1 catalytic domain with an added HA-tag was ordered from MrGene and cloned into *Hind*III and *Xho*I sites of pcDNA3 (Invitrogen). The clone was validated by sequencing. The coding sequence of *A. mellifera* 5-methylcytosine dioxygenase catalytic domain was PCR amplified with Go-Taq-Pfu DNA polymerase cocktail (Promega) and cloned into *Kpn*I and *Xho*I sites of pcDNA3 (Invitrogen) with an HA-tag added. The clone was validated by sequencing. In order to obtain a catalytically inactive AmTET, residues H264 and D266 responsible for iron coordination were mutated to tyrosine and alanine, respectively (numbering refers to the cloned ORF).

### 5.2. HEK293T transfection

HEK293T cells were maintained according to the ATCC protocol. Cells were seeded in 6-well plates and at the 70% of confluence were transfected with 1.5 μg of pAmTET plasmid or with a mixture of 0.5 μg GFP and 1 μg of empty plasmids. Transfection was done using polyethyleneimine (Sigma) for 48 h and after 2 h the proteasome inhibitor clasto-lactacystin β-lactone (Cayman Chemical) was added (5 µM) followed by 16 h of additional growth. After transfection, the cells were washed twice with PBS and used for downstream analysis. Both methods gave the same results. The transfection efficiency was 30–40%.

### 5.3. Western blot

The following primary antibodies were used for an overnight incubation at 4°C: rabbit anti-HA (1 : 1000, Sigma) and rabbit anti-GAPDH (1 : 1000, Santa Cruz Biotechnology). The secondary anti-rabbit IgG antibody, conjugated with horseradish peroxidase (Sigma), was applied for 45 min at room temperature. Blots were visualized with enhanced chemiluminescence (ECL) and exposed onto an X-ray film.

### 5.4. DNA dot-blot

Genomic DNA (gDNA) was isolated by phenol–chloroform extraction. Samples were diluted in 0.1 NaOH, heated to 95°C, spotted onto a positively charged nylon membrane (Pall), dried and cross-linked with UV light for 4 min. The membrane was blocked in PBS–Tween 20 buffer with 10% non-fatty milk, and then was incubated with anti-5hmC rabbit antibody (1 : 5000, Active Motif) overnight at 4°C. The secondary anti-rabbit IgG antibody, conjugated with horseradish peroxidase (Sigma), was applied for 45 min at room temperature. Blots were visualized with ECL and an ImageQuant LAS4000 imager (GE Healthcare). Densitometry was performed with the dedicated software.

### 5.5. Immunofluorescence staining

Cells were fixed with 4% PFA and permeabilized with 0.1% Triton for 10 min. DNA was denaturated with 12% HCl for 10 min and then neutralized with 100 mM Tris pH 8.5 for 15 min. Afterwards, the cells were washed twice with PBS, blocked using 5% BSA and incubated with primary antibodies: rabbit anti-HA (1 : 1000, Sigma) overnight in 4°C, and mouse anti-5hmC (1 : 500, Active Motif) for 2 h at room temperature (RT). The secondary anti-rabbit IgG antibody conjugated with Alexa Fluor 647 and anti-mouse IgG with Alexa Fluor 568 (both from Molecular Probes) were applied for 45 min at RT. Slide images were acquired with a Zeiss LSM5 Exciter confocal microscope.

### 5.6. Thin layer chromatography 5hmC pulldown

Honeybee genomic DNA (20 µg), 20 µg of phage lambda dam^−^ dcm^−^ DNA (Thermo Scientific), 4 µg of mouse brain DNA and 80 ng of 5hmC PCR product (with all cytosines replaced with 5hmC) were digested overnight with DNase I at 37°C. After degradation, 200 µl of Tris-buffered saline with Tween-20 (TBST) and 5 µl of mouse monoclonal antibody mAb (Active Motif) were added to the solution. Samples were incubated for 3 h in RT with gentle agitation. Protein G magnetic beads (10 μl; Merck), treated with anti-mouse IgG bridging antibody (Active Motif), were added to the solution and incubated for 1 h at RT. Beads were washed four times with TBST and twice with ddH_2_O. Afterwards, the samples were suspended in Degradase (Zymo) buffer and denatured for 20 min at 85°C. 5U of Degradase (Zymo) was added to each sample and incubated for 2 h at 37°C. Reactions were stopped by incubation at 80°C for 10 min. Obtained single nucleotides were first dephosphorylated with FastAP (Thermo Scientific) and then labelled with P32 gamma ATP (Hartmann Analytic) using T4 PNK (Thermo Scientific). Samples were resolved on PEI-cellulose TLC plates (Merck) and extended in 66 : 20 : 1 isobutyric acid : H_2_O : NH_4_. Results were visualized by exposing the plate onto an X-ray film.

### 5.7. 5-Hydroxymethylcytosine quantification using ^3^H-UDP-glucose

A modification of the method described by Szwagierczak *et al.* [34] was used. Labelling reactions were carried out in NEB buffer 4 (NEB: 50 mM potassium acetate; 20 mM Tris–acetate; 10 mM magnesium acetate: 1 mM DTT). 4U of T4 phage β-glucosyltransferase; 1 nM UDP-[^3^H] glucose (glucose-6-^3^H; 60 μCi mmol^−1^; Hartmann Analytic) and 2–4 µg of sample DNA were used for each reaction. Reactions were incubated for 1 h at 37°C followed by a 20 min heat-inactivation at 65°C. Afterwards, the reactions were spotted onto DEAE cellulose, washed five times with Tris-buffered saline, Tween 20, EDTA and once with 70% ethanol. Remaining radioactivity was measured using a Tri-Carb 2900TR liquid scintillation counter (Packard) in Rotiszint Eco Plus scintillation liquid (Roth). The 5hmC fraction of total cytosine was calculated using a calibration curve obtained from labelling a PCR product with dCTP swapped for d5hmCTP. Our estimate of the number of 5mhCs per haploid genome is based on the genome size of 260 Mb [35,36].

### 5.8. Molecular biology methods

Extraction and processing of nucleic acids was performed using our established protocols [16,17]. *In situ* hybridization and developmental stage evaluation was described earlier [20,21]. Brain anatomy nomenclature is based on reference [37]. Primers for amplification of AmTET catalytic domain were: forward (F): AAAGGTACCGAAATGGATTACCCATACGATGTTCCAGATTACGCTGAAGTGCCGGACTGCAACTGCTTC; reverse (R): GGCCTCGAGTCATCCAATGGCACCTCCCTCCTGA. Primers for qPCR-AmTET: F1: GTCAGTGAGATCAGAGGAGC; R1: TGGTGCAAGGCTGAGGTACA; for AmDNMT3: F1: TACAAACTGTCGGAGGTGCA; R1: AGCGTCGTCCAAAGTCCAGT; for AmTET fragment used for *in situ* hybridization: F2: GACGAATTCGGACTTGTTACTACA; R2: GCGAAGCTTGATCGTTGTAGACTTGTTGCT. See more details in the electronic supplementary material.

### 5.9. Transcript variants level estimation

RNAseq reads from the GenBank SRA database were queried with 120 bp long sequences covering symmetrically all predicted exon 4 3′ splice junctions using stand-alone BLAST+. Specific junctions were identified and scored by analysing the resulting alignments; a score was incremented if there was a continuous (ungapped) alignment of minimum 70 nucleotides. Transcript content is estimated as a percentage of a specific junction in all junctions analysed.

AmTET reads in RNAseq datasets were identified as follows. Gene-specific read numbers were extracted from RNAseq alignments to *Apis* Genome assembly v. 4.5 generated in BAM format using SAMtools (www.samtools.sourceforge.net) and used for transcript expression level calculations. Genome assembly v. 4.5 is available via www.beebase.org. RNAseq data are available from the GenBank SRA database or from our server via a guest login (contact RM for details).

### 5.10. Biological sample collection and fixation

Adult bees were collected from our Canberra colonies. Queens were purchased from local beekeepers. Eggs were collected from a small artificial comb (Karl Jenter, Nürtingen, Germany) attached to a wooden frame hosting a confined queen that was allowed to lay for 4 h. Following the laying period, the queen was removed, and the egg-containing frame was transferred to an incubator at 35°C, 80% humidity for later collection. Larvae were harvested from brood frames taken from the hive and incubated at 35°C, 80% humidity and snap frozen in liquid nitrogen if required. Dissections of the queen ovaries and drone genitals were carried out in a standard bee Ringer solution.

## Supplementary Material

Figure S1

## Supplementary Material

Figure S2

## Supplementary Material

Figure S3

## Supplementary Material

Figure S4
