# Supplementary material for: Insights into DNA hydroxymethylation in the honeybee from in-depth analyses of TET dioxygenase
Source: Open Biol. 2014 Aug 6;4(8):140110. doi: 10.1098/rsob.140110 (PMC4150289; doi:10.1098/rsob.140110)
Supplement: Figure S2 [file rsob140110supp2.pdf]

Figure S2. AmTET and AmDNMTs transcripts levels in RNA seq datasets.
See legend to figure S1 for more details

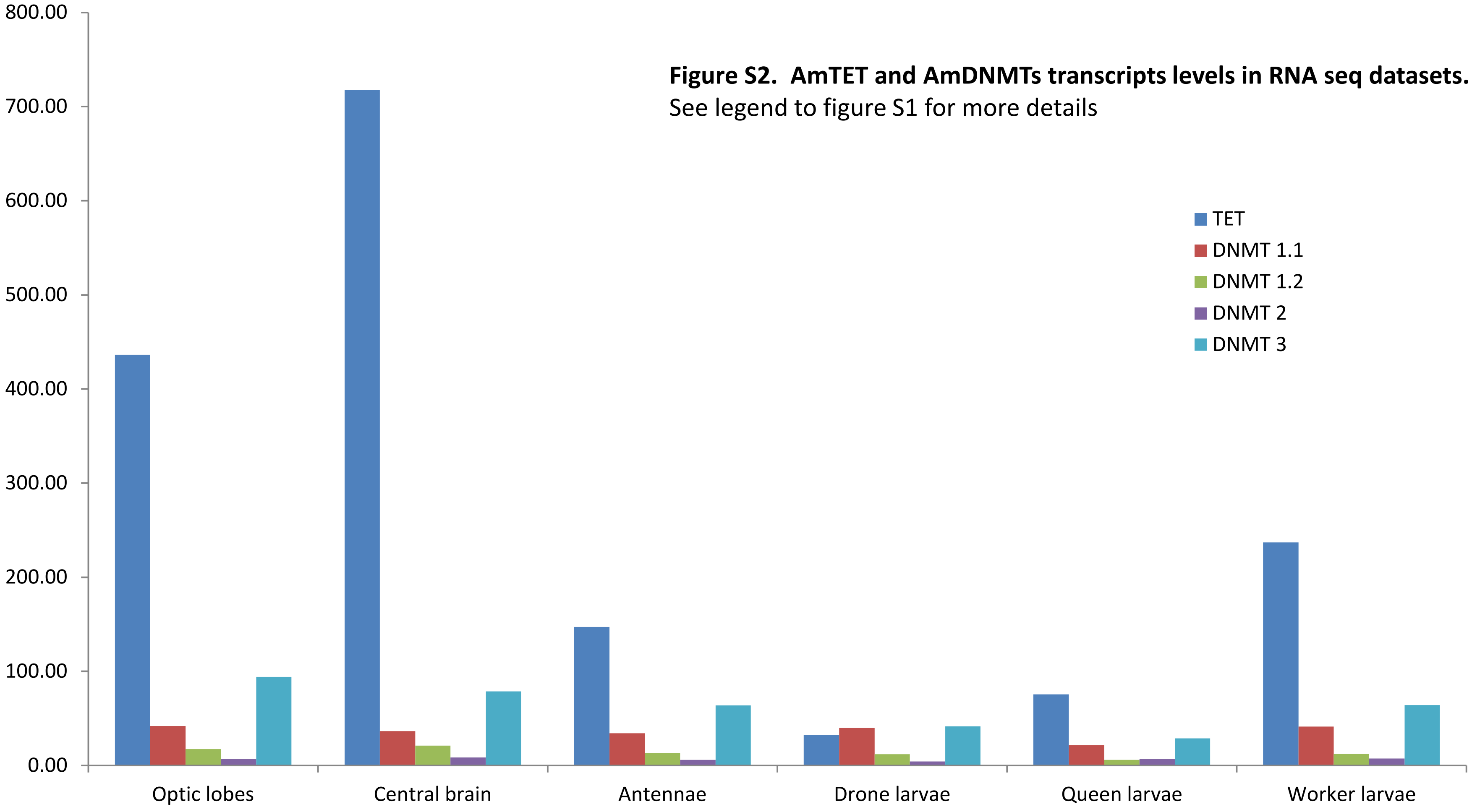

apm	Optic lobes	Central brain	Antennae	Drone larvae	Queen larvae	Worker larvae
TET	436.40	717.92	147.10	32.41	75.44	236.81
Dnmt1.1	41.81	36.57	34.28	39.88	21.66	41.36
Dnmt1a	17.39	21.09	13.38	12.03	5.80	12.21
Dnmt2	7.02	8.51	5.87	4.31	7.06	7.31
Dnmt3	94.04	78.65	63.97	41.57	28.66	63.99

STDEV	Optic lobes	Central brain	Antennae	Drone larvae	Queen larvae	Worker larvae
TET	45.87	36.75	nd	1.24	7.20	10.20
Dnmt1.1	6.28	5.05	nd	2.85	1.83	3.94
Dnmt1a	3.41	3.14	nd	1.04	0.65	1.87
Dnmt2	2.15	1.18	nd	1.34	1.59	1.64
Dnmt3	4.77	4.01	nd	2.72	2.40	4.61
N	4	4	1	5	5	5
