# Supplementary material for: Insights into DNA hydroxymethylation in the honeybee from in-depth analyses of TET dioxygenase
Source: Open Biol. 2014 Aug 6;4(8):140110. doi: 10.1098/rsob.140110 (PMC4150289; doi:10.1098/rsob.140110)

Figure S3. qPCR analysis of AmTET and AMDNMT3 experssion in various honey bee samples. See material and methods for more details.

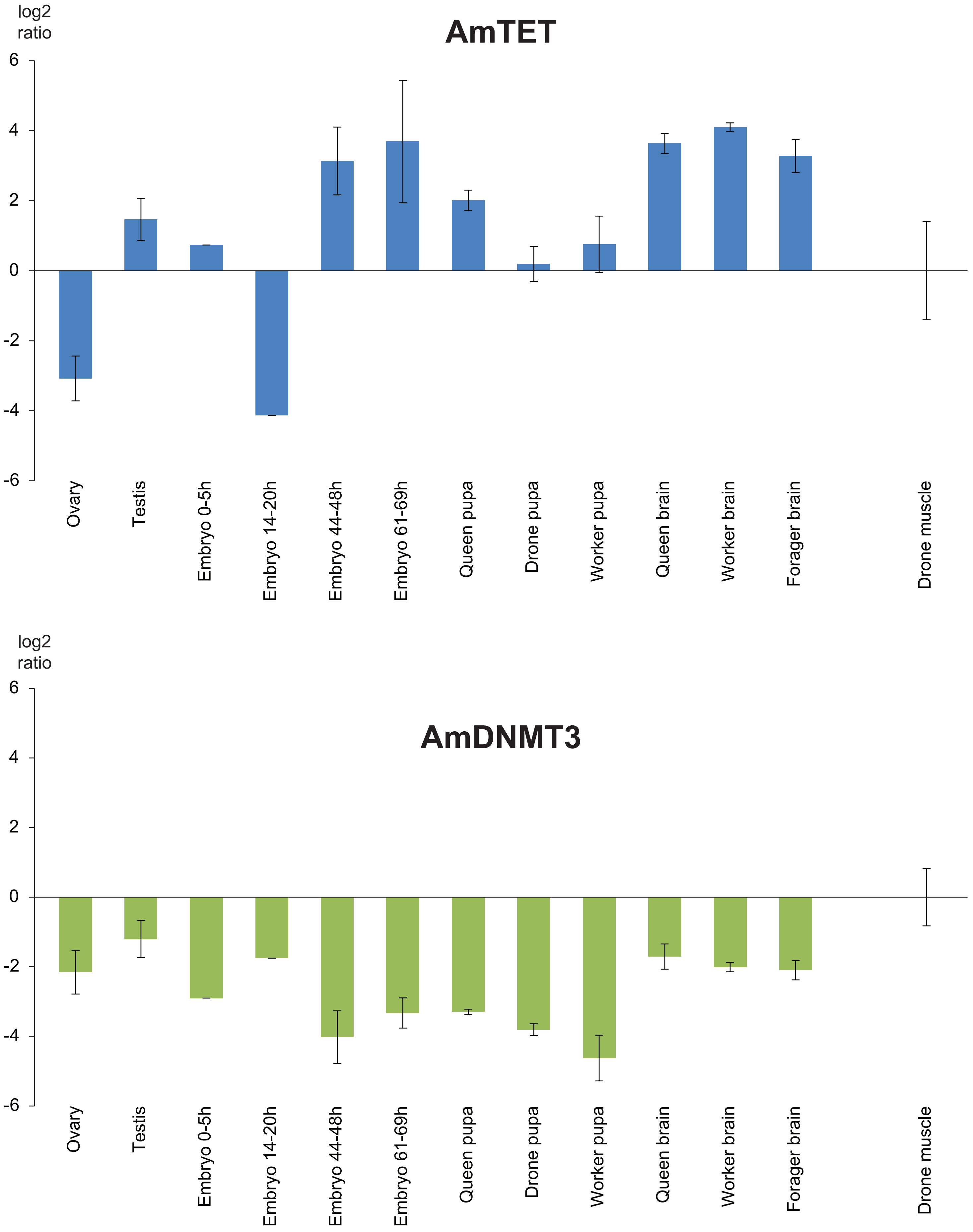

Supplement: Figure S3 [file rsob140110supp3.pdf]
