# Supplementary material for: Insights into DNA hydroxymethylation in the honeybee from in-depth analyses of TET dioxygenase
Source: Open Biol. 2014 Aug 6;4(8):140110. doi: 10.1098/rsob.140110 (PMC4150289; doi:10.1098/rsob.140110)
Supplement: Figure S1 [file rsob140110supp1.pdf]

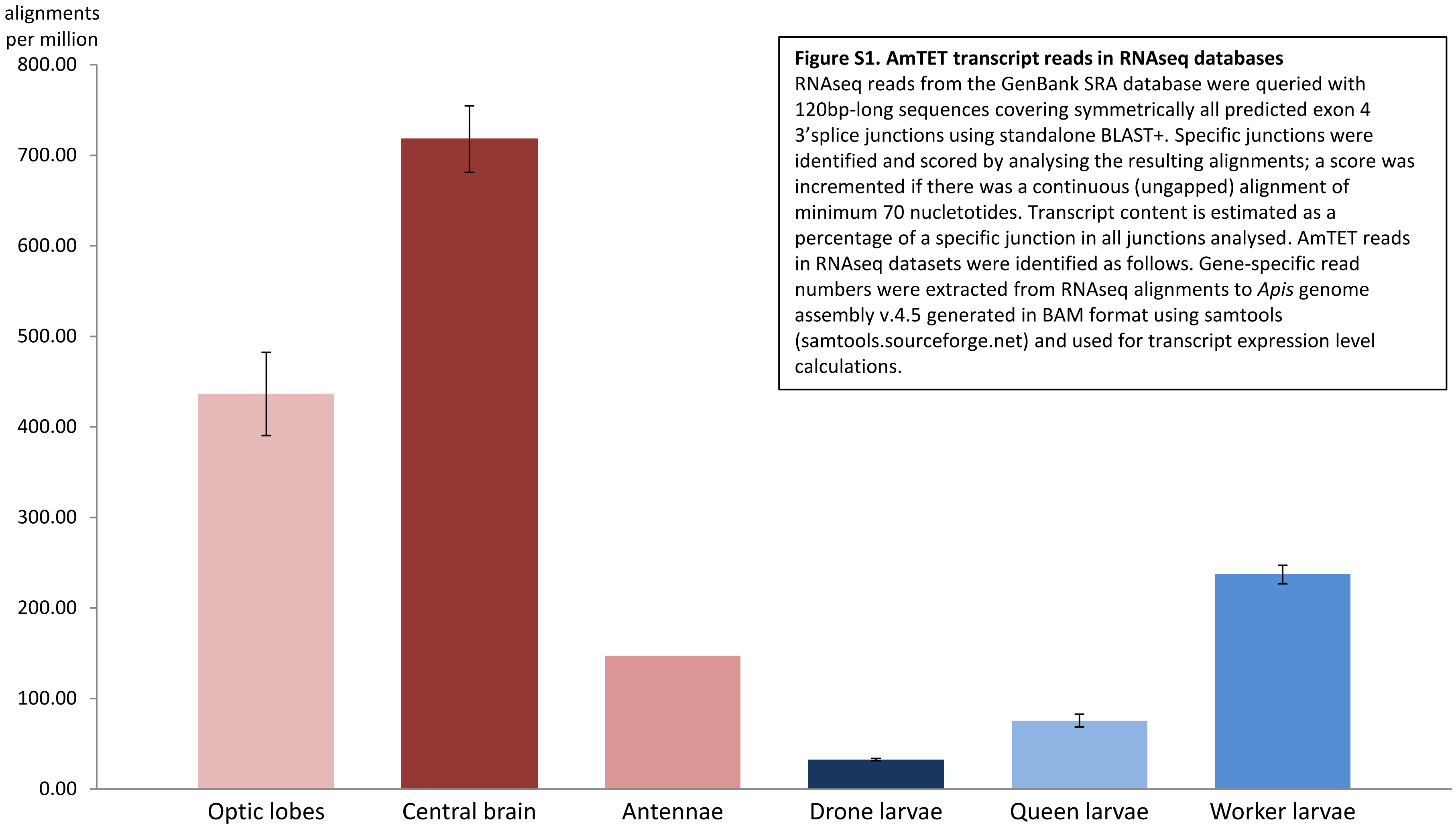

Figure S1. AmTET transcript reads in RNAseq databases
RNAseq reads from the GenBank SRA database were queried with 120bp-long sequences covering symmetrically all predicted exon 4 3'splice junctions using standalone BLAST+. Specific junctions were identified and scored by analysing the resulting alignments; a score was incremented if there was a continuous (ungapped) alignment of minimum 70 nucletotides. Transcript content is estimated as a percentage of a specific junction in all junctions analysed. AmTET reads in RNAseq datasets were identified as follows. Gene-specific read numbers were extracted from RNAseq alignments to *Apis* genome assembly v.4.5 generated in BAM format using samtools (samtools.sourceforge.net) and used for transcript expression level calculations.

AmTET GB52555	Optic lobes	Central brain	Antennae	Drone larvae	Queen larvae	Worker larvae
apm	436.40	717.92	147.10	32.41	75.44	236.81
stdev	45.87	36.75	nd	1.24	7.20	10.20
N	4	4	1	5	5	5
apm= alignments per million						
stdev =standard deviation; N = number of experiments						
