# Supplementary material for: Insights into DNA hydroxymethylation in the honeybee from in-depth analyses of TET dioxygenase
Source: Open Biol. 2014 Aug 6;4(8):140110. doi: 10.1098/rsob.140110 (PMC4150289; doi:10.1098/rsob.140110)
Supplement: Figure S4 [file rsob140110supp4.pdf]

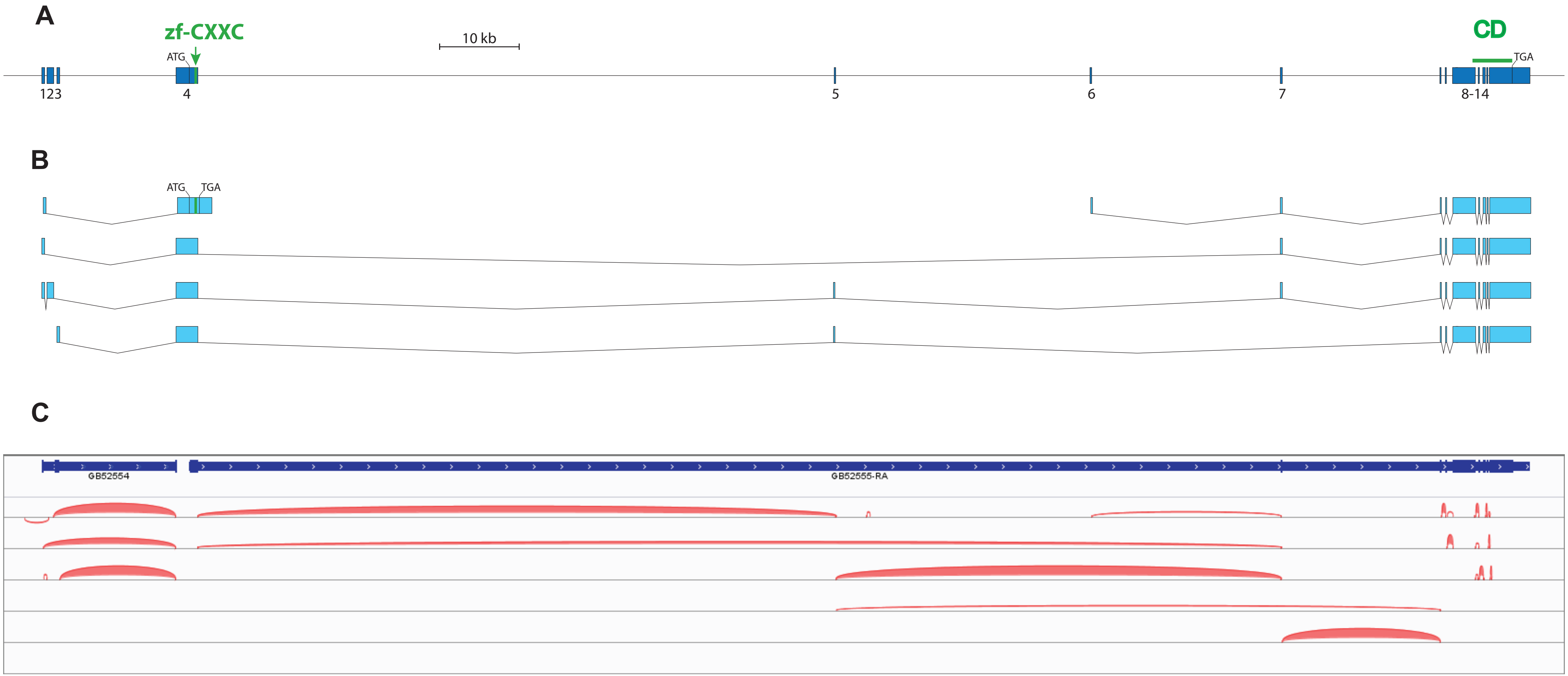

Figure S4
A. AmTET gene model showing all detected exons, zf-CXXC: DNA binding domain; CD: catalytic domain
B. Transcript models based on RNAseq data
C. Spliced junctions (RNA-seq/tophat). Integrative Genomics Viewer (IGV) visualisation of splice junctions in a typical RNA-seq sample from brains identified via tophat/cufflinks reads analysis pipeline (www.broadinstitute.org/igv/). References: Thorvaldsdóttir et al.(2013)Integrative Genomics Viewer (IGV): high-performance genomics data visualization and exploration. *Brief Bioinform*. 14: 178-192, and Robinson et al (2011)Integrative Genomics Viewer. *Nature Biotech*. 29:24–26.
